# Exploring the Role of Chemokine Receptor 6 (*Ccr6*) in the BXD Mouse Model of Gulf War Illness

**DOI:** 10.3389/fnins.2020.00818

**Published:** 2020-08-14

**Authors:** Jun Gao, Fuyi Xu, Athena Starlard-Davenport, Diane B. Miller, James P. O’Callaghan, Byron C. Jones, Lu Lu

**Affiliations:** ^1^Department of Genetics, Genomics, and Informatics, University of Tennessee Health Science Center, Memphis, TN, United States; ^2^Institute of Animal Husbandry and Veterinary Science, Shanghai Academy of Agricultural Sciences, Shanghai, China; ^3^Health Effects Laboratory Division, Centers for Disease Control and Prevention, National Institute for Occupational Safety and Health, Morgantown, WV, United States

**Keywords:** *Ccr6*, GWI, DFP, CORT, BXD strain, RNA-seq, neuroinflammation

## Abstract

Gulf War illness (GWI) is a chronic and multi-symptomatic disorder with persistent neuroimmune symptomatology. Chemokine receptor 6 (CCR6) has been shown to be involved in several inflammation disorders in humans. However, the causative relationship between *CCR6* and neuroinflammation in GWI has not yet been investigated. By using RNA-seq data of prefrontal cortex (PFC) from 31 C57BL/6J X DBA/2J (BXD) recombinant inbred (RI) mouse strains and their parental strains under three chemical treatment groups – saline control (CTL), diisopropylfluorophosphate (DFP), and corticosterone combined with diisopropylfluorophosphate (CORT+DFP), we identified *Ccr6* as a candidate gene underlying individual differences in susceptibility to GWI. The *Ccr6* gene is *cis*-regulated and its expression is significantly correlated with CORT+DFP treatment. Its mean transcript abundance in PFC of BXD mice decreased 1.6-fold (*p* < 0.0001) in the CORT+DFP group. The response of *Ccr6* to CORT+DFP is also significantly different (*p* < 0.0001) between the parental strains, suggesting *Ccr6* is affected by both host genetic background and chemical treatments. Pearson product-moment correlation analysis revealed 1473 *Ccr6*-correlated genes (*p* < 0.05). Enrichment of these genes was seen in the immune, inflammation, cytokine, and neurological related categories. In addition, we also found five central nervous system-related phenotypes and fecal corticosterone concentration have significant correlation (*p* < 0.05) with expression of *Ccr6* in the PFC. We further established a protein-protein interaction subnetwork for the *Ccr6-*correlated genes, which provides an insight on the interaction of G protein-coupled receptors, kallikrein-kinin system and neuroactive ligand-receptors. This analysis likely defines the heterogeneity and complexity of GWI. Therefore, our results suggest that *Ccr6* is one of promising GWI biomarkers.

## Introduction

Gulf War illness (GWI) is the term used to describe a chronic and multi-symptomatic disorder affecting returning military veterans of the 1990–1991 Gulf War (Binns et al., 2014). The symptoms of GWI vary somewhat among individuals and typically include unexplained fatigue, chronic diarrhea musculoskeletal pain, headaches, cognitive dysfunction, rashes and respiratory problems, gastrointestinal, and dermatologic complaints (White et al., 2016; Maule et al., 2018). Although some views ascribed GWI to post-traumatic stress disorder (PTSD) or psychiatric condition to the consequence of wars, accumulated evidence shows that GWI is a neuroimmune disorder resulting from chemical exposures and the physiological stressors incurred in the war theater (O’Callaghan et al., 2015; White et al., 2016). Animal model behavioral data mirror GWI neurobehavioral deficits in terms of impaired memory and cognition, as well as increased anxiety and depressive-like mood (Abdullah et al., 2011; Parihar et al., 2013; Hattiangady et al., 2014; Zakirova et al., 2015; Carreras et al., 2018; Carpenter et al., 2020).

The neurotoxicant exposures encountered by GW military personnel during deployment, including carbamates, organophosphates (OPs), and other pesticides; OP nerve agents (sarin/cyclosarin); and pyridostigmine bromide (PB) (White et al., 2016; Maule et al., 2018). Accumulated neuroimaging studies have demonstrated abnormalities in the brains of veterans with GWI (Binns et al., 2014) including strong evidence for neuroinflammation (Alshelh et al., 2020). Brain pathology of reduced white and gray matter volumes also can be detected nearly two decades later in sarin and cyclosarin-exposed ill GW veterans (Chao et al., 2011). Studies revealed that brain chemistry is abnormal mainly in prefrontal cortex (PFC) and different subregions that mediate various characteristics of the chronic pain, such as sensory and affective dimensions, anxiety and depression (Apkarian et al., 2005). Changes in neurotransmitters, gene expression, glial cells, and neuroinflammation occur in the PFC during acute and chronic pain, which result in alterations to its structure, activity, and connectivity (Ong et al., 2019). Moreover, cortical regions involved in fatigue, pain, and hyperalgesia, also have been reported to be associated with diminished white matter integrity in GW veterans (GWV) (Rayhan et al., 2013). However, heterogeneous symptom presentation and lack of biomarkers in PFC that identify a distinct pathophysiological process in GWI still remain challenging.

Chronic inflammation is a component of the pathophysiology of GWI (Johnson et al., 2016). The sarin surrogate diisopropylfluorophosphate (DFP), an irreversible acetylcholinesterase (AChE) inhibitor, results in brain-wide neuroinflammation that is markedly enhanced in the mouse model by prior exposure to CORT (O’Callaghan et al., 2015; Locker et al., 2017; Koo et al., 2018). High circulating glucocorticoids exaggerates the neuroinflammatory response as measured by the expression of genes for multiple cytokines and chemokines (e.g., *Tnf-α*, *Il6*, *Ccl2*, *Il-1β*, *Lif*, and *Osm*) (O’Callaghan et al., 2015; Jones et al., 2020). Neuroinflammation disorder is induced by chemical exposure and has been linked to cytokine-induced ‘sickness’ behavior of GWI in veterans (Dantzer and Kelley, 2007; Dantzer et al., 2008; O’Callaghan et al., 2015); however, the underlying causes have not been fully elucidated.

Chemokine receptor 6 (CCR6) contributes to steady-state cell chemotaxis in supporting immunity and regulating immune homeostasis during inflammation (Ranasinghe and Eri, 2018). Genetic associations have been identified between *CCR6* polymorphisms and immune system disorders in humans including rheumatoid arthritis (RA) and Crohn’s disease (Cheng et al., 2015; Julian et al., 2017). GWI is also characterized by gastrointestinal disorders such as inflammatory bowel disease (IBD) like Crohn’s disease (Ranasinghe and Eri, 2018; Seth et al., 2019). In addition, RA is reported to overlap with specific druggable components of GWI, and some immunosuppressants have been approved by the Food and Drug Administration (FDA) as the best available candidates for treating GWI symptoms (Craddock et al., 2015). However, the causative relation between *CCR6* and GWI has not been reported yet.

The C57BL/6J X DBA/2J (BXD) recombinant inbred (RI) mouse strains, which are unique mosaic of alleles derived from the parental C57BL/6J (B6) and DBA/2J (D2) strains have been constructed as a high precision genetic reference population for systems genetics in unraveling the genetic architecture of polygenic traits (Ashbrook et al., 2019). The BXD family consists of more than 150 BXD fully inbred strains that segregate for ∼6 million genetic variants and thus can be used as an informative murine genetic reference panel. The application of the BXD strains provides a unique mouse model to investigate the role of *Ccr6* in individual differences to GWI susceptibility.

In this study, we assessed the expression of *Ccr6* in the PFC of the GWI BXD model with different chemical treatments. Furthermore, we sought to identify the eQTL for *Ccr6*, analyze correlated genes and potential pathways, and to construct a protein-protein interaction (PPI) subnetwork that may contribute to individual differences in GWI.

## Materials and Methods

### Animals

Four hundred-nine mice from 31 BXD strains and their parental strains (B6 and D2) were used in this study. The animals were randomly chosen at 2–4 months of age at testing and 2–3 animals per strain, sex and treatment group were used (Supplementary Data 1). All animals were housed in individually ventilated cage (IVC) system in the Animal Care Facility at the University of Tennessee Health Science Center (UTHSC, Memphis, TN, United States). The vivarium is a temperature (20 ± 2°C) and humidity (35%) controlled environment under a 12 h light/12 h dark cycle. The animals had free access to food and water throughout the experiment. Nine days before the euthanasia, every mouse was single caged, and received corresponding treatment after 2 days adaptation. The euthanasia was carried out in a separate procedure room. All animal procedures were carried out in accordance with the UTHSC guidelines on the humane treatment of experimental animals and with the explicit approval of the Institutional Animal Care and Use Committee (IACUC).

### Treatment Groups

The experimental animals were divided into three treatment groups (Jones et al., 2020) as follows:

(1) Control group (**CTL**): These strains received plain tap water for fluid (Day 1–7). On the 8th day, the animals were injected with saline (0.9% NaCl) and euthanized by cervical dislocation 6 h after injection.
(2) Diisopropylfluorophosphate group (**DFP**): These strains received plain tap water for fluid (Day 1–7). On the 8th day, the animals were injected with 4 mg/kg DFP, i.p., 6 h after injection, the animals were euthanized by cervical dislocation followed by decapitation.
(3) Corticosterone + Diisopropylfluorophosphate group (**CORT+DFP**): These strains received tap water containing 20 mg% CORT dissolved in 0.6% (v/v) EtOH vehicle for 8 days. On the 8th day, the animals were injected with 4mg/kg DFP, i.p., 6 h after injection, the animals were euthanized by cervical dislocation followed by decapitation.

The chemicals DFP (Sigma, St. Louis, MO, United States), CORT (Steraloids, Inc., Newport, RI, United States) and other reagents were analytical grade.

### Tissue Collection

The mice were euthanized by cervical dislocation followed by decapitated that is descripted in our previous publication (Jones et al., 2020) and the whole brain was immediately removed from the skull. The PFC was dissected with a 90° cut 1 mm from the posterior edge of olfactory bulb and another 90° cut 2 mm caudal from the first cut. The PFC was weighed and snap frozen in dry ice bath with isopentane and stored at −80°C until RNA extraction.

### RNA-Seq and Data Processing

Total RNA was extracted from 20 mg frozen PFC tissue per sample using RNeasy Mini Kit (Qiagen) according to the manufacturer’s instructions. The concentration and purity of the RNA was measured using NanoDrop spectrophotometer (Thermo Fisher Scientific, Wilmington, DE, United States). The RNA integrity (RIN) was assessed using Agilent 2100 Bioanalyzer (Agilent, Santa Clara, CA, United States). 1 mg qualified RNA (per sample) with OD260/280 > 1.8, OD260/230 > 2.0, and RNA Integrity Number (RIN) >8.0 was used for library preparation and sequencing. The RNA-seq libraries were prepared using the NEBNext^®^ Ultra RNA Library Prep Kit at Novogene Corporation Inc. Paired-end sequencing was performed on an Illumina Novaseq Platform (Illumina, San Diego, CA, United States) by reading 150 bases at each end of a fragment. Overall, each library generated an average of 40 million raw reads.

Raw reads, stored in fastq format, were filtered by removing the adaptor and low-quality reads for further analysis. To generate clean reads, allowing for reads containing over 50% bases with quality greater than 5 and less than 10% “N” bases to be included. The clean reads were then mapped onto the mouse reference genome (version: GRCm38) using the STAR aligner (v2.5.0a) (Dobin et al., 2013). FeatureCount (v0.6.1) (Liao et al., 2014) program was used to get the gene level reads count based on the gene model annotation file downloaded from the Ensembl genome browser^1^. Raw read count was normalized by DESeq2 R package (v1.22.2) (Love et al., 2014) and batch was added as a covariate for data normalization. Differential expression of *Ccr6* was calculated between the three groups (DFP vs. CTL, CORT+DFP vs. CTL, and DFP vs. CORT+DFP) by unpaired *t*-test.

### Analysis of Variance (ANOVA)

A two between-subjects variables (strain, treatment) design was used to assess the main effects and interaction on *Ccr6* transcript abundance using ANOVA function in R software (R Core Team, 2013). The accepted level of significance for all tests was *p* < 0.05.

### Heritability Estimation

Broad sense heritability (*h*^2^) is a concept that summarizes how much of the variance in a quantitative trait is due to variation in genetic factors. It was calculated from the ANOVA results using the following formula (Hegmann and Possidente, 1981): 0.5 VA/(0.5 VA + VE), where VA is the additive genetic variance (variances of the strain means) and VE is the average environmental variance (variance within strains). The factor of 0.5 in this formula was applied to adjust for the 2-fold increase in the additive genetic variance among the inbred strains relative to outbred populations (Lu et al., 2018).

### eQTL Mapping and Sequence Variants Analysis

eQTL mapping is a regression analysis to determine the relationship between differences in a trait and differences in alleles at markers across the genome. The eQTL mapping of *Ccr6* in three groups (CTL, DFP, and CORT+DFP) were conducted through the WebQTL module on GeneNetwork website^2^ according to the published methods (Mulligan et al., 2017; Williams and Williams, 2017). The input expression values of *Ccr6* was normalized with TPM (transcripts per million) method (Wagner et al., 2012; Vera Alvarez et al., 2019) and log2 (TPM + 1) transformed. Simple interval mapping yielded a likelihood ratio statistic (LRS) score, providing us a quantitative measure of confidence of linkage between the observed phenotype and a genomic region. The genome-wide significance (*p* < 0.05) for each eQTL was determined with 1000 permutation tests.

Single nucleotide polymorphisms (SNPs) and insertion-deletions (InDels) in the *Ccr6* gene and its surrounding up- and down-stream regions between the B6 and D2 were extracted from the Mouse Genome Project database^3^ (Keane et al., 2011; Yalcin et al., 2011).

### Gene-Phenotype Correlation Analysis

To assess the relationship between the expression of *Ccr6* and related traits across the BXD cohort, we queried the BXD archival phenotypes from the GeneNetwork and analyzed for Pearson product-moment correlation to the expression of *Ccr6* in PFC. The top 500 Pearson product-moment correlations were filtered and *p* < 0.05 were considered significant.

### Gene-Gene Correlation Analysis

In order to identify the *Ccr6* correlated genes across the PFC transcriptomes in the treatment groups, we conducted Pearson product-moment correlations of the strain means between the expression of *Ccr6* and the expression of all the other genes across the mouse genome to produce sets of genetically correlated genes on GeneNetwork. Genes significantly correlated with expression of *Ccr6* (*p* < 0.05) were used for the gene set enrichment analysis, in which, Riken cDNA clones, intragenic sequences, and predicted genes were eliminated.

### Gene Set Enrichment Analysis

Gene set enrichment analysis was performed to investigate the gene ontology (GO, biological processes) and Kyoto Encyclopedia of Genes and Genomes (KEGG) pathway of the *Ccr6* correlated genes. We submitted the gene set of each treatment group to the Webgestalt website^4^ (Liao et al., 2019) for analysis. The *p*-value generated from the test was automatically adjusted to account for multiple comparisons using the Benjamini and Hochberg correction (Benjamini and Hochberg, 1995). A minimum overlap of five genes and False Discovery Rate (FDR) < 0.05 was required to determine the genes significantly overrepresented in those categories.

### Protein-Protein Interactions (PPI) Analysis

PPI analysis of the *Ccr6* correlated genes was based on the online STRING (Search Tool for the Retrieval of Interacting Genes/Proteins) database (Szklarczyk et al., 2019), which contains known and predicted PPIs information by consolidating known and predicted protein-protein association data for a large number of organisms (Szklarczyk et al., 2016). In this study, we first constructed the PPI network by extracting the target gene lists from the database with required the highest score of confidence interaction of 0.9. Then Markov Cluster Algorithm (MCL) clustering was used for subnetwork construction, in which the inflation parameter used a default setting 3. The narrowed subnetwork genes were further used for GO and KEGG analysis to gain insight into the biological functions and pathways of *Ccr6* correlated genes.

## Results

### *Ccr6* Expression Across the BXD Strains

**FIGURE 1:**
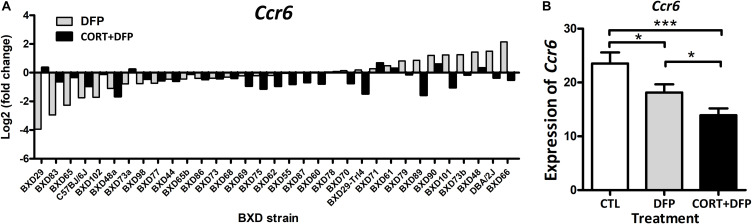
The expression of *Ccr6* across the BXD strains. **(A)** The relative fold change of *Ccr6* expression in the DFP and CORT+DFP groups compared to the CTL across the BXD RI strains. **(B)** Comparison of the expression (Mean ± SEM) of *Ccr6* between the treatments (CTL, DFP, and CORT+DFP) by unpaired *t*-test. *p < 0.05, ***p < 0.0001. The *Ccr6* expression is a normalized value by the DEseq2.

In this study, a total of 409 mice were used for PFC harvest and expression profiling across the treatments. Overall, the expression of *Ccr6* in CORT+DFP group showed significant decrease in most of the BXD strains (Figure 1A). However, this effect was not consistent for DFP treatment, in which *Ccr6* mRNA levels decreased in 18 strains (e.g., BXD29, BXD83, BXD65), but increased in the rest of 15 strains (e.g., BXD66, D2, BXD48). This finding further supports the assertion of O’Callaghan et al. (2015) that exposure to OPs plus high circulating glucocorticoids may be an essential condition for GWI. Next, we compared the expression of *Ccr6* between the different treatment groups. Results showed that *Ccr6* significantly decreased in the CORT+DFP group when compared with CTL group (Fold change = 1.60, *p* < 0.0001) and DFP group (Fold change = 1.42, *p* < 0.05) (Figure 1B), respectively.

In order to determine the effects of strain and treatment on the expression of *Ccr6* further, we conducted two way ANOVA which showed both factors have significant effects on the *Ccr6* expression [Treatment: *F*_(2, 310)_ = 22.20, *p* < 10E-10; Strain: *F*_(32, 310)_ = 18.15, *p* < 3E-16]. The strain × treatment interaction was also significant [*F*_(64, 310)_ = 2.40, *p* < 4.0E-7]. In addition, we calculated the heritability (*h*^2^) for each treatment group with *h*^2^ = 0.29 for CTL, 0.27 for DFP and 0.22 for CORT+DFP, suggesting both genetic and environmental factors contribute to the expression differences of *Ccr6* among the BXD strains.

### eQTL Mapping and Sequence Variants of *Ccr6*

**FIGURE 2:**
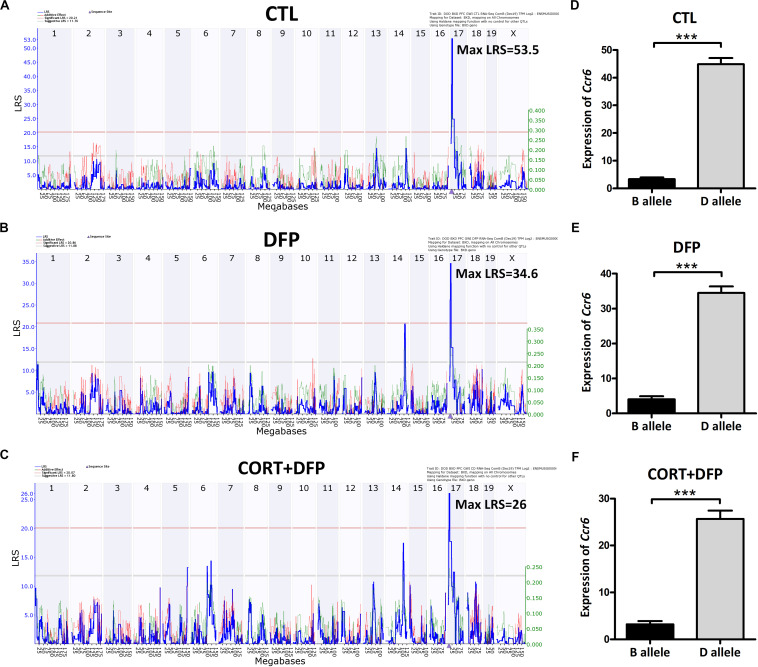
eQTLs mapping of *Ccr6.* eQTLs mapping demonstrates *Ccr6* is *cis* regulated in the prefrontal cortex mRNA. **(A)** CTL (Max likelihood ratio statistic score (LRS) = 53.5), **(B)** DFP (Max LRS = 34.6), **(C)** CORT+DFP (Max LRS = 26.0). Chromosome number can be found across the top of the plot with megabases (Mb) on the x-axis. The y-axis contains the LRS. The location of *Ccr6* is marked by an arrowhead found on the x-axis is at Chr 17 at 8.236 Mb. **(D–F)** The expression (Mean ± SEM) of *Ccr6* is significantly different between B and D allele by unpaired *t*-test, ***p < 0.0001. The *Ccr6* expression is a normalized value by the DEseq2.

*Ccr6* is located on chromosome 17 at 8.236 Mb of mice. Interval mapping indicated a genome-wide significant eQTL with a LRS of 53.5 in CTL (Figure 2A), 34.6 in DFP (Figure 2B), and 26 in CORT+DFP (Figure 2C) on chromosome 17 at 7.713 Mb. This locus is located 0.5 megabases (Mb) upstream of *Ccr6*, indicating that *Ccr6* is *cis*-regulated in the PFC for all three groups (Figure 2). Next, we grouped the mice according to their genotype (B and D type) at the QTL peak position (rs48543649, Chr 17 at 8.199 Mb) which is near the physical position of *Ccr6*. Statistical analysis revealed that the mRNA levels of *Ccr6* showed a significant difference (*p* < 0.0001) between B and D alleles in all three treatment groups (Figures 2D–F), with mice carrying the D allele evincing higher expression level of *Ccr6*.

*Ccr6* is *cis* regulated, which means that sequence variants within or nearby *Ccr6* likely affect its expression. Therefore, we explored nearby genetic variants using the database of Mouse genome project^6^. We identified 31 SNPs and 3 InDels (Table 1) between the parental strains B6 and D2, in which one is a synonymous variant (rs49056705), three are 5^ˊ^ UTR variants (rs33886456, rs33640330, and rs33573638), and the rest of them are located within 5000 bp upstream of *Ccr6*. We also identified one trans-eQTL achieved statistical significance in DFP group, which located on Chr14 at 100–110 Mb. This eQTL interval contains 90 genes, of which, *Kctd12* and *Mycbp2* correlated with *Ccr6* (*p* < 0.05), *Slain1* and *Ednrb* harbor nonsynonymous mutations, suggesting they could be upstream candidate regulators.

**TABLE 1:** Genetic variants of the *Ccr6* gene between B6 and D2 strain.

**Chr**	**Position**	**Gene**	**dbSNP**	**B6**	**D2**	**Location**
17	8237775	*Ccr6*	rs33886505	C	T	Upstream
17	8238900	*Ccr6*	rs33887421	C	T	Upstream
17	8238918	*Ccr6*	rs33886574	G	A	Upstream
17	8238921	*Ccr6*	rs33886578	G	A	Upstream
17	8239193	*Ccr6*	rs108936048	G	T	Upstream
17	8239196	*Ccr6*	rs46526090	T	A	Upstream
17	8239353	*Ccr6*	rs50262179	G	A	Upstream
17	8239675	*Ccr6*	rs108682332	C	T	Upstream
17	8239968	*Ccr6*	rs51108167	T	C	Upstream
17	8240151	*Ccr6*	rs33887529	C	A	Upstream
17	8240159	*Ccr6*	rs33887367	C	T	Upstream
17	8240202	*Ccr6*	rs33886450	C	T	Upstream
17	8240541	*Ccr6*	rs33886337	C	T	Upstream
17	8240545	*Ccr6*	rs33886772	A	G	Upstream
17	8240816	*Ccr6*	rs33886245	A	G	Upstream
17	8241148	*Ccr6*	rs33887345	A	G	Upstream
17	8241192	*Ccr6*	rs33886743	T	G	Upstream
17	8241200	*Ccr6*	rs33886642	A	G	Upstream
17	8241364	*Ccr6*	rs33887184	T	C	Upstream
17	8241391	*Ccr6*	rs33886254	T	G	Upstream
17	8241601	*Ccr6*	rs33887415	T	C	Upstream
17	8241739	*Ccr6*	rs33886977	T	C	Upstream
17	8241990	*Ccr6*	rs108371987	G	c	Upstream
17	8242244	*Ccr6*	rs33886406	A	G	Upstream
17	8242597	*Ccr6*	rs33886456	G	A	5^ˊ^ UTR
17	8242718	*Ccr6*	rs108834476	T	C	Upstream
17	8242869	*Ccr6*	rs108768852	T	A	Upstream
17	8243414	*Ccr6*	rs33886432	A	G	Upstream
17	8255770	*Ccr6*	rs33640330	G	A	5^ˊ^ UTR
17	8255795	*Ccr6*	rs33573638	T	C	5^ˊ^ UTR
17	8256696	*Ccr6*	rs49056705	A	C	Synonymous
17	8237780	*Ccr6*	rs252574186*	AC	A	Upstream
17	8242367	*Ccr6*	rs252004148*	C	CGCTGACAGAGG	Upstream
17	8242872	*Ccr6*	rs254441880*	C	CT	Upstream

31 SNPs and 3 InDels (*asterisk marked) variants were identified between the B6 and D2 strains from the Database of Mouse genome project (https://www.sanger.ac.uk/science/data/mouse-genomes-project). “Chr” indicates the Chromosome. Upstream variants are located within 5000 bp upstream of the gene.

### Genetic Correlations Between *Ccr6* and Archival Phenotypes From Our Database in GeneNetwork Website

**FIGURE 3:**
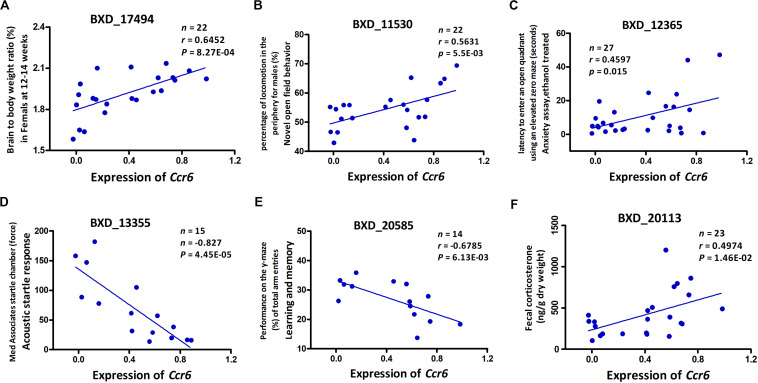
Phenotype Pearson correlation analysis with the expression of *Ccr6*. Five BXD central nervous system (CNS) related phenotypes including brain to body weight ratio **(A)**, novel open field behavior **(B)**, anxiety assay **(C)**, acoustic startle response **(D)**, and learning and memory **(E)** were significantly correlated with the expression of *Ccr6* in PFC. The fecal CORT content was positively correlated with the expression of *Ccr6* **(F)**. GeneNetwork BXD phenotype identifiers (e.g., ID “BXD_17494”) are at the top of each plot. *n*, Number of strains, *p*, *p*-value.

To our knowledge, GWI is a chronic disease with significant neurological pathophysiology. In our mouse model, exposure to CORT+DFP treatment increased expression of proinflammatory cytokine genes, which is consistent with the neuroimmune basis of GWI (O’Callaghan et al., 2015). Additionally, our results show that CORT+DFP has the greatest effect on the expression of *Ccr6*. The question then becomes does CORT+DFP-related expression of *Ccr6* associate with other central nervous system (CNS) phenotypes? After multiple testing correction (FDR < 0.05), we obtained a total of 117 CNS related phenotypes that were significantly correlated with the expression of *Ccr6* in the CORT+DFP group (*P* < 0.05) (Supplementary Data 2). We listed five CNS-related phenotypes in BXD RI strains, including brain to body weight ratio (Figure 3A), novel open field behavior (Figure 3B), anxiety assay (Figure 3C), acoustic startle response (Figure 3D), learning and memory (Figure 3E), as well as fecal corticosterone concentration (Figure 3F) significantly correlated (*p* < 0.05) with the expression of *Ccr6*. These correlated phenotypes can be found on the GeneNetwork website with the access numbers of 17494, 11530, 12365, 13355, 20585, and 20113, respectively.

### Gene Set Enrichment Analysis

To understand the biological processes and gene pathways of *Ccr6* correlated genes, we performed correlation analysis and identified 1755, 7193, and 3996 genes that are significantly correlated (*p* < 0.05) with *Ccr6* in the CTL, DFP, and CORT+DFP group, respectively. After removing Riken cDNA clones, intragenic sequences, predicted genes, 806 (CTL), 3983 (DFP), and 1473 (CORT+DFP) genes were separately submitted to Webgestalt web site^7^ for gene function enrichment analysis.

**FIGURE 4:**
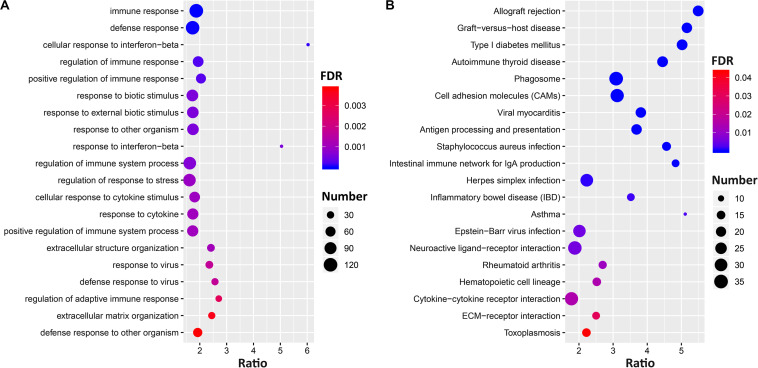
The gene set enrichment analysis of *Ccr6* correlated genes. The bubble plot shows the top 20 GO **(A)** and KEGG pathways **(B)** of *Ccr6* correlated genes in the CORT+DFP group.

The gene set enrichment results (Supplementary Data 3) showed *Ccr6* correlated genes were significantly enriched in immune and inflammation-related GO terms in the CTL group. For the DFP group, the enrichment results demonstrate a high degree of neurological association with the *Ccr6* correlated genes. For the CORT+DFP group, we obtained a total of 47 significantly enriched GO terms (FDR < 0.05) and 23 KEGG pathways (FDR < 0.05). The top 20 GO and KEGG categories are listed in Figure 4. Of which, immune, inflammation, and cytokine terms were further highlighted.

### Protein-Protein Interactions (PPI) Subnetwork for *Ccr6* Correlated Genes

**FIGURE 5:**
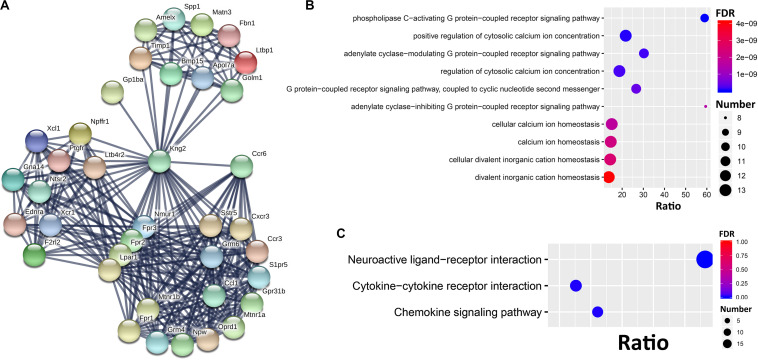
PPI subnetwork of *Ccr6* correlated genes. **(A)** Ccr6 PPI subnetwork includes 38 genes with the interaction score ≥0.9. The nodes represent genes while edges represent PPIs between two genes. Bubble plot of the Top 10 GO **(B)** and 3 KEGG **(C)** enrichment categories for the 38 PPI subnetwork genes.

To further dissect the potential interactions of the *Ccr6* correlated genes in the CORT+DFP group, we uploaded the above 1473 genes correlated to *Ccr6* into STRING^5^ to search for PPI. By performing MCL clustering, we identified a Ccr6 PPI subnetwork, which includes 38 genes (Figure 5). These genes are highly inter-connected (interaction score ≥ 0.9).

Next, we performed GO and KEGG enrichment analysis for these 38 genes. The top 10 significant GO terms (FDR < 0.05) showed that those genes are mainly involved in G protein-coupled receptor signaling pathways (Oprd1, Ednra, Nmur1, Ntsr2, Fpr1, F2rl2, Lpar1, Fpr3, Gna14, Fpr2, Grm4, Sstr5, Grm6, Ptgfr, Cxcr3, and Mtnr1a) and cytosolic calcium ion concentration (Xcl1, Ednra, Fpr1, Ccl1, Ccr3, Ccr6, F2rl2, Lpar1, Ptgfr, Fpr3, Kng2, and Fpr2) (Figure 5B). The top 10 KEGG pathways (Figure 5C) had 3 achieve significance (FDR < 0.05), including one neuroactive ligand-receptor interaction pathway (Ltb4r2, Oprd1, Npffr1, S1pr5, Ednra, Grm4, Nmur1, Sstr5, Ntsr2, Fpr1, F2rl2, Grm6, Lpar1, Ptgfr, Mtnr1b, Fpr3, Mtnr1a, and Fpr2), and two cytokine-related pathways (Xcl1, Ccl1, Ccr3, Ccr6, Bmp15, Cxcr3, and Xcr1).

## Discussion

Compared to nondeployed veterans, at least one fourth of the 697,000 U.S. veterans suffered from GWI when they returned from the theater of operations (Binns et al., 2014). These veterans also reported higher rates of amyotrophic lateral sclerosis (ALS) (Coffman et al., 2005; Horner et al., 2008), brain cancer (Barth et al., 2009), repeated seizures, neuralgia or neuritis, stroke (Kang et al., 2009), and migraine headaches (Unwin et al., 1999; Kang et al., 2000; Steele, 2000; Gray et al., 2002). Accumulating Studies clearly supports the links between adverse neurological outcomes and chemical exposures of GWV (Binns et al., 2014; White et al., 2016). Deployed GWV had significantly lower scores on tests of verbal memory, verbal learning, motor speed, and attention than nondeployed due to the pesticides and PB exposures (Toomey et al., 2009). Sarin/cyclosarin exposed GWV showed signs of reduced total gray and white matter volumes in the brain compared to unexposed controls and worse on a continuous performance test of attention (Chao et al., 2011).

Accurate diagnosis and treatment of GWI patients require an in-depth understanding of the cause of the disease. Although some of the individual differences in susceptibility to GWI may be explained by different exposures or different dose effects, much of it cannot be, and leaves genetics as a significant contributor to individual differences in susceptibility and response to the exposures. The BXD mouse strains put the investigator at great advantage for systems genetics analysis of complex traits such as GWI and those traits that have modest heritability (Williams et al., 2001). In this study, we used BXD strains to explore the etiologic agents and pathways that underlie the “sickness” behavior of GWI. Indeed overall, we have supported the notion that this disease is the result of genetic–environment interaction.

Our results indicate *Ccr6* as one reasonable candidate gene that underlies individual differences in susceptibility to GWI. In addition, we also found five CNS-related phenotypes that show the wide-ranging effects of GWI. Furthermore, we identified 31 SNPs and 3 InDels that differ in response to CORT+DFP between B6 and D2 inbred strains. *CCR6* may turn out to be a target of therapeutic approaches to GWI.

The results of gene set enrichment analysis highlighted the categories of *Ccr6* correlated genes related to immune, inflammation, cytokine, and neurological aspects. Accumulating evidence suggest that *Ccr6* plays a major role in driving T-helper differentiation in inflammatory diseases and maintaining leukocyte homeostasis (Ranasinghe and Eri, 2018). *Ccr6* regulates the migration of inflammatory and regulatory T cells (Th17 and Treg), which play opposite roles in autoimmune diseases (Yamazaki et al., 2008). Although *Ccr6*-mediated Th17 migration to inflamed tissues may be important for driving CNS inflammation, *Ccr6* expression is deemed to be more critical to Treg cells than to Th17 cells, because this subset suppresses inflammatory T cell proliferation and promotes disease resolution (Ranasinghe and Eri, 2018). The use of *Ccr6*^–/^*^–^* mice in experimental autoimmune encephalomyelitis (EAE) study, an animal model of brain inflammation for the study of human CNS diseases characterized by mononuclear cell infiltration and demyelination, showed delayed disease onset and more neurological damage and increased mortality compared to wild-type mice (Villares et al., 2009). Severe phenotype in *Ccr6*^–/^*^–^* EAE mice is linked to increased inflammatory activity in target tissues. This suggests that *Ccr6* is necessary for Treg recruitment and initiates a feedback anti-inflammatory mechanism which compensatorily downregulates the CNS inflammatory activity (Yamazaki et al., 2008; Villares et al., 2009). Although the immune regulation mechanism of *CCR6* has not been fully elucidated, the *CCR6/CCL20* axis is an important chemokine receptor-ligand and may present a therapeutic target for the treatment of human disorders (Ranasinghe and Eri, 2018).

As we understand, GWI is a complex trait with underlying gene-environment and likely gene-gene interactions. Indeed, we identified a Ccr6 PPI subnetwork that includes 38 genes enriched in G protein-coupled receptor (GPCR) signaling pathways and cytosolic calcium ion concentration signaling pathway. Astrocytes and microglia are the most prominent target cells for inflammation in the CNS. Their responses upon activation include downregulation of ATP-induced Ca^2+^ signaling, G protein activities and release of pro-inflammatory cytokines (Hansson et al., 2018). Many druggable targets for treatment of common diseases involve GPCRs that mediate therapeutic effects of ∼34% of the marketed drugs (Hauser et al., 2018). Further understanding of genetic factors and regulation networks of *Ccr6* is likely to advance drug treatment of GWI in the future.

Another gene worth noting is Kng2, that appears as a hub in the Ccr6 PPI subnetwork, implicating that the Kallikrein-kinin system (KKS) mediate the pathophysiological features of neurological disorders, including GWI. Despite a paucity of literature on Kng2, pharmacological research in mice and human genetic analyses suggest that the KKS may regulate anxiety (Nokkari et al., 2018; Rouhiainen et al., 2019). On the other hand, *Ccr6* correlated genes in PFC were observed to be significantly enriched in neuroactive ligand-receptor interaction pathways, of which, three formylpeptide receptors (Fpr1, Fpr2, Fpr3) are critical mediators of myeloid cell trafficking in microbial infection, inflammation, and immune responses (Krepel and Wang, 2019). Fpr2 also proved to mediate anxiety as shown by effects reported for Fpr agonists (Zhao et al., 2016). Although the role of the Ccr6 PPI subnetwork genes and their contribution to the neuroinflammation of GWI are not fully elucidated, it provides a new insight to the complexity of GWI.

### Limitations

A suitable animal model of GWI should show evidence of the illness acutely and have it persist to model the entire 30-year course of the symptoms exhibited by ill veterans. The data in the present manuscript models the acute condition. We also have an extension of this model that represents the chronic “primed” inflammatory condition (manuscript in internal review). The chronic model is based on the paradigm of systemic challenge with lipopolysaccharide (LPS) (Kelly et al., 2018). It is important to subsequently investigate the changes of *Ccr6* mRNA levels at later time points in the chronic model to verify the possibility that *CCR6* can be a marker for GWI or similar ailment. Given the complexity and heterogeneity of GWI, we cannot exclude the existence of other regulatory elements. For example, genes such as *Tnf-α*, *Il6*, *Il1β*, and *Spon1* may also be involved in the neuroinflammatory response of GWI (Jones et al., 2020).

## Conclusion

In this study, we identified *Ccr6* involvement in the neuroimmune response to CORT+DFP treatment in the BXD mouse model of GWI. Genetic factors and treatments both impact on the expression of *Ccr6* in PFC, which may contribute to CORT+DFP neuroinflammation in BXD strains. In humans, *CCR6*-mediates the migration of inflammatory and regulatory T cells and regulates CNS inflammation, which indicates it may be a promising therapeutic target of GWI. Our study also suggests the polymorphisms of *Ccr6* and synergy interaction of the related GPCRs, KKS system, and neuroactive ligand-receptor may contribute to the heterogeneity and complexity of GWI and related sickness behaviors.

## Data Availability Statement

The datasets generated for this study can be found in the online repositories. The names of the repository/repositories and accession number(s) can be found below: www.genenetwork.org, GN880 (http://gn1.genenetwork.org/webqtl / main. py ? FormID = sharinginfo &GN _AccessionId = 880 ), GN881 (http://gn1.genenetwork.org/webqtl/main.py?FormID= sharinginfo&GN_AccessionId=881), and GN882 (http://gn1. genenetwork . org /w ebqtl / main . py ? FormID = sharinginfo & GN _AccessionId=882).

## Ethics Statement

The animal study was reviewed and approved by the Institutional Animal Care and Use Committee (IACUC).

## Author Contributions

LL, BJ, DM, and JO’C conceived the study and oversaw the execution of the experimental work. JG and FX performed data analysis and prepared the figures and tables. LL, JG, and FX wrote the manuscript. BJ, LL, JO’C, and AS-D edited the manuscript. All authors read and approved the final version of the manuscript.

## Disclaimer

The findings and conclusions in this report are those of the authors and do not necessarily represent the official position of the National Institute for Occupational Safety and Health, Centers for Disease Control and Prevention. This work was supported by the Assistant Secretary of Defense for Health Affairs, through the Gulf War Illness Research Program. Opinions, interpretations, conclusions and recommendations are those of the authors and are not necessarily endorsed by the Department of Defense.

## Conflict of Interest

The authors declare that the research was conducted in the absence of any commercial or financial relationships that could be construed as a potential conflict of interest.

## Footnotes

1 https://useast.ensembl.org/

2 www.genenetwork.org

3 http://www.sanger.ac.uk/science/data/mouse-genomes-project

4 http://www.webgestalt.org

5 https://string-db.org/

6 https://www.sanger.ac.uk/science/data/mouse-genomes-project

7 http://www.webgestalt.org

## Supplementary Material

DATA S1 Sample information.

DATA S2 *Ccr6* correlated phenotypes in BXD mice.

DATA S3 Enrichment results of *Ccr6* correlated genes.
